# Supplementary material for: Architecture and regulation of a GDNF-GFRα1 synaptic adhesion assembly
Source: Nat Commun. 2023 Nov 20;14:7551. doi: 10.1038/s41467-023-43148-8 (PMC10661694; doi:10.1038/s41467-023-43148-8)
Supplement: Supplementary file 1 — Supplementary Information [file 41467_2023_43148_MOESM1_ESM.pdf]

SUPPLEMENTARY INFORMATION

Architecture and regulation of a GDNF-GFRα1 synaptic adhesion assembly

Houghton, FM[1], Adams SE^[1], Ríos AS[2], Masino, L[3], Purkiss AG[3], Briggs DC[1], Ledda, F[2], & McDonald N.Q.[1,4,*]

[1] Signalling and Structural Biology laboratory, The Francis Crick Institute, 1 Midland Road, London, NW1 1AT, United Kingdom.
[2] Fundación Instituto Leloir, Instituto de Investigaciones Bioquímicas de Buenos Aires, Av. Patricias Argentinas 435, C1405BWE, Buenos Aires, Argentina.
[3] Structural Biology Science and Technology Platform, The Francis Crick Institute, 1 Midland Road, London, NW1 1AT, United Kingdom.
[4] Institute of Structural and Molecular Biology, Department of Biological Sciences, Birkbeck College, Malet Street, London, WC1E 7HX, United Kingdom.

Current address: ^ Vertex Pharmaceuticals, 86-88 Jubilee Avenue, Milton Park, Abingdon, Oxfordshire OX14 4RW, United Kingdom.

* Corresponding author, Signalling and Structural Biology laboratory, The Francis Crick Institute, 1 Midland Road, London, NW1 1AT, United Kingdom.

SUPPLEMENTARY FIGURES

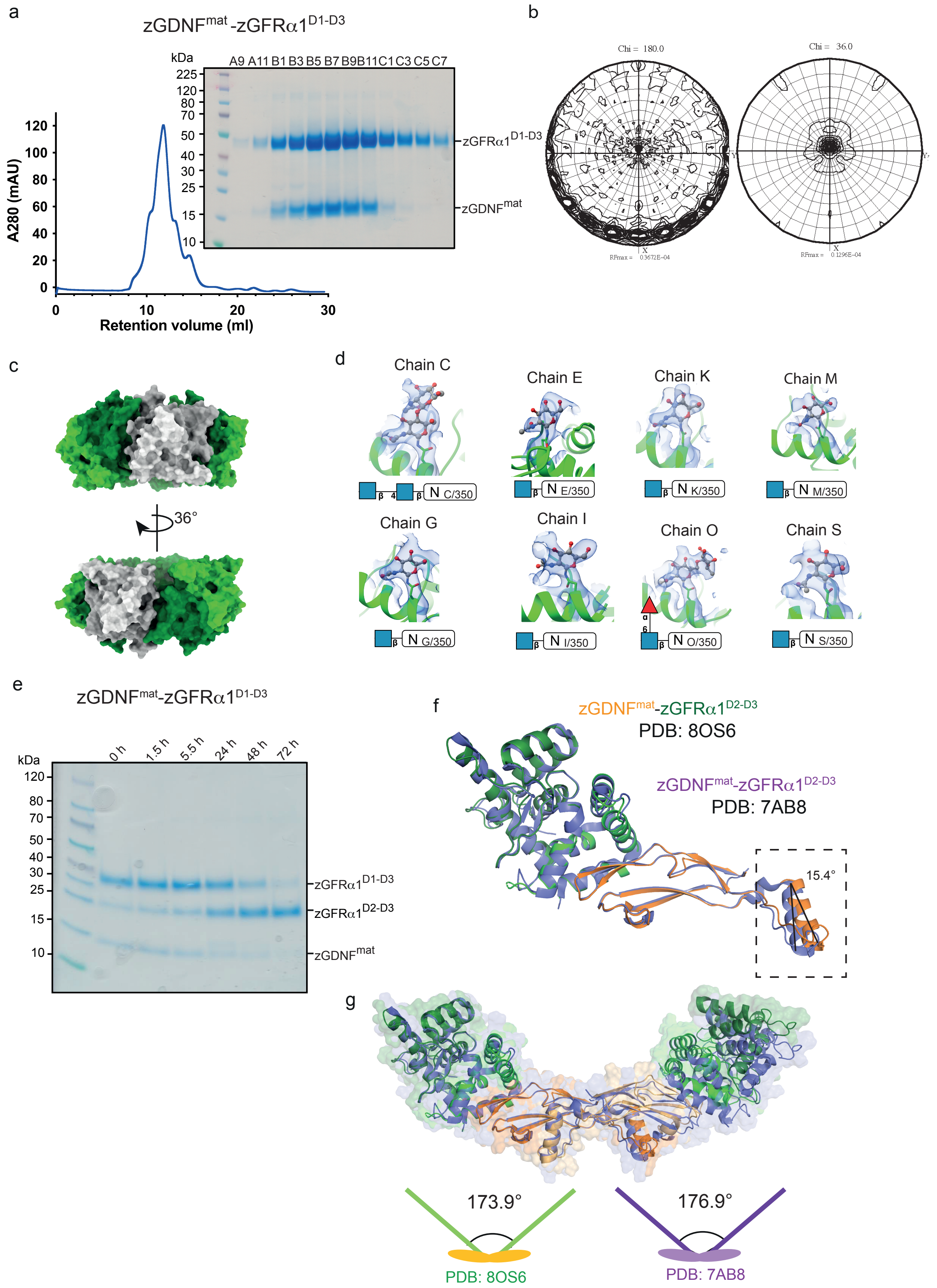

Supplementary Fig. 1. Purification, crystallographic and structural analysis of zGDNF^{mat} -zGFRα1^{D1-D3} multimers. (a) SEC profile of zGDNF^{mat}-zGFRα1^{D1-D3} run on a Superdex 200 10/300 column. SDS-PAGE gel of peak fractions is shown within the inset. **(b)** Self-rotation function (SRF) analysis of the 30 – 5Å diffraction data from the zGDNF^{mat}-zGFRα1^{D1-D3} crystal. Left: The Chi = 180° section of the SRF with a total of 10 peaks indicating ten 2-fold axes and Right: The Chi = 36° section of the SRF with a single peak indicating a 5-fold non-crystallographic symmetry axis. Self-rotation analysis was calculated and visualized with MOLREP in CCP4I[1,2]. **(c)** The two zGFRα1 pentamers from the zGDNF^{mat}-zGFRα1^{D1-D3} 10:10 crystal structure are shown as a surface representation, with a single zGFRα1 protomer from each subunit coloured in grey. This highlights the 36° rotation around the 5-fold axis between the two sub-complexes. **(d)** Asparagine (N)-linked glycans within the zGDNF^{mat}-zGFRα1^{D2-D3} structure. Final electron density map (m2F_O-DF_c difference electron density map) contoured to 1σ at each N-linked glycosylation site with the modelled N-linked glycans superposed. The backbone of (i) zGFRα1 and (ii) zGDNF are shown in cartoon representation and the attached glycans as sticks. Schematics of modelled glycoforms were generated by PRIVATEER[3]. **(e)** SDS-PAGE analysis of proteolytic clipping time-course for the zGFRα1 D1 domain. Purified zGDNF^{mat} -zGFRα1^{D1-D3} was incubated for indicated timepoints at 30 °C and the extent of D1 clipping assessed by the generation of a new lower molecular weight band running at 25 kDa. **(f)** Structural superimposition of a single zGDNF^{mat} -zGFRα1^{D2-D3} 1:1 assembly from the decameric complex (PDB: 8OS6) with the 1:1 zGDNF^{mat} -zGFRα1^{D2-D3} structure previously published (PDB: 7AB8) Conformational differences are seen in the relative angle of the helical structural element of GDNF, with a 15.4° rotation between the two helical elements. **(g)** Structural superimposition of a 2:2 zGDNF^{mat}-zGFRα1^{D2-D3} assembly from the decameric complex, and the 2:2 assembly of zGDNF^{mat}-zGFRα1^{D2-D3} previously published (PDB: 7AB8). Inter-protomer bend angles were calculated in PyMOL[4] by measuring the angle between two ß-finger elements and a central disulfide bridge, Glu162Cα–Cys202Sγ–Glu162Cα'[5].

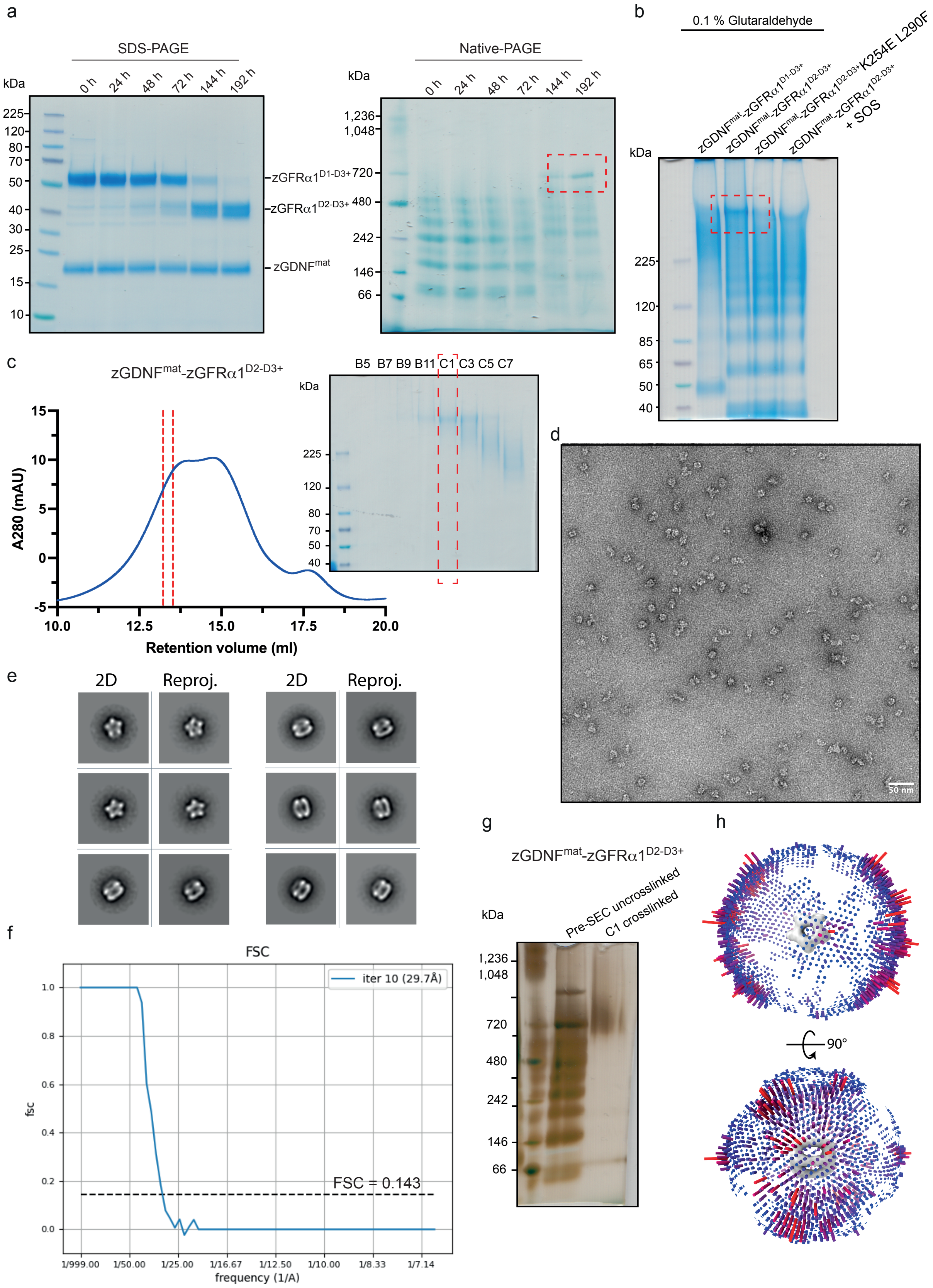

Supplementary Fig. 2. Biochemical and negative-stain EM validation of the zGDNF-zGFRα1 decameric assembly. (a) Reducing SDS-PAGE gel (i) and native-PAGE gel (ii) of purified zGDNF^{mat}-zGFRα1^{D1-D3+}. Purified zGDNF^{mat} -zGFRα1^{D1-D3+} was incubated for indicated timepoints at 30 °C and the extent of D1 clipping assessed

by the generation of a new lower molecular weight band running at 40 kDa. By 192 h, almost complete cleavage of the D1 domain has occurred. A high molecular weight band (~700kDa), indicated by the red box, consistent with the decameric complex, can be observed at 192 h by native-PAGE formed from a 2:2 complex in solution upon proteolytic clipping of the N-terminal D1 domain. **(b)** Reducing Tris-Acetate SDS-PAGE gel of crosslinked recombinant zGDNF^{mat}-zGFRα1 samples. zGFRα1 recombinant samples in complex with zGDNF^{mat} indicated above the gel. The red box indicates a higher molecular weight band > 225 kDa observed for zGDNF^{mat}-zGFRα1$^{D2\text{-}D3+}$ sample. **(c)** SEC profile of crosslinked zGDNF^{mat}-zGFRα1$^{D2\text{-}D3+}$ and reducing SDS-PAGE gel of fractions across the elution peak. The red dashed box indicates the gel-filtration fraction, C1, applied to EM grids and negatively stained (NS). **(d)** Representative NS-EM micrograph of purified crosslinked zGDNF^{mat}-zGFRα1$^{D2\text{-}D3+}$, from a total of 540 micrographs **(e)** Projection matching performed using xmipp3 "compare reprojection" protocol[6] between the RELION (v3.1)[7,8] 2D class averages from the particles used to generate the final 3D reconstruction, and different reprojections of the 3D model. **(f)** Fourier shell correlation (FSC) curve of the zGDNF^{mat} -zGFRα1$^{D1\text{-}D3+}$ NS-EM map. Resolution reported based on the gold-standard FSC threshold, FSC = 0.143, shown as a dashed line. **(g)** Native-PAGE gel of zGDNF^{mat}-zGFRα1$^{D2\text{-}D3+}$ prior to crosslinking (left-hand lane) and post crosslinking and SEC purification (right-hand lane). **(h)** The orientation distribution of particles that contribute to the final reconstruction of the zGDNF^{mat}-zGFRα1$^{D2\text{-}D3+}$ decameric complex. The predominant view is parallel to the five-fold molecular dyad (side view), and a fraction of particles viewed perpendicular to the 5-fold rotational symmetry (top view).

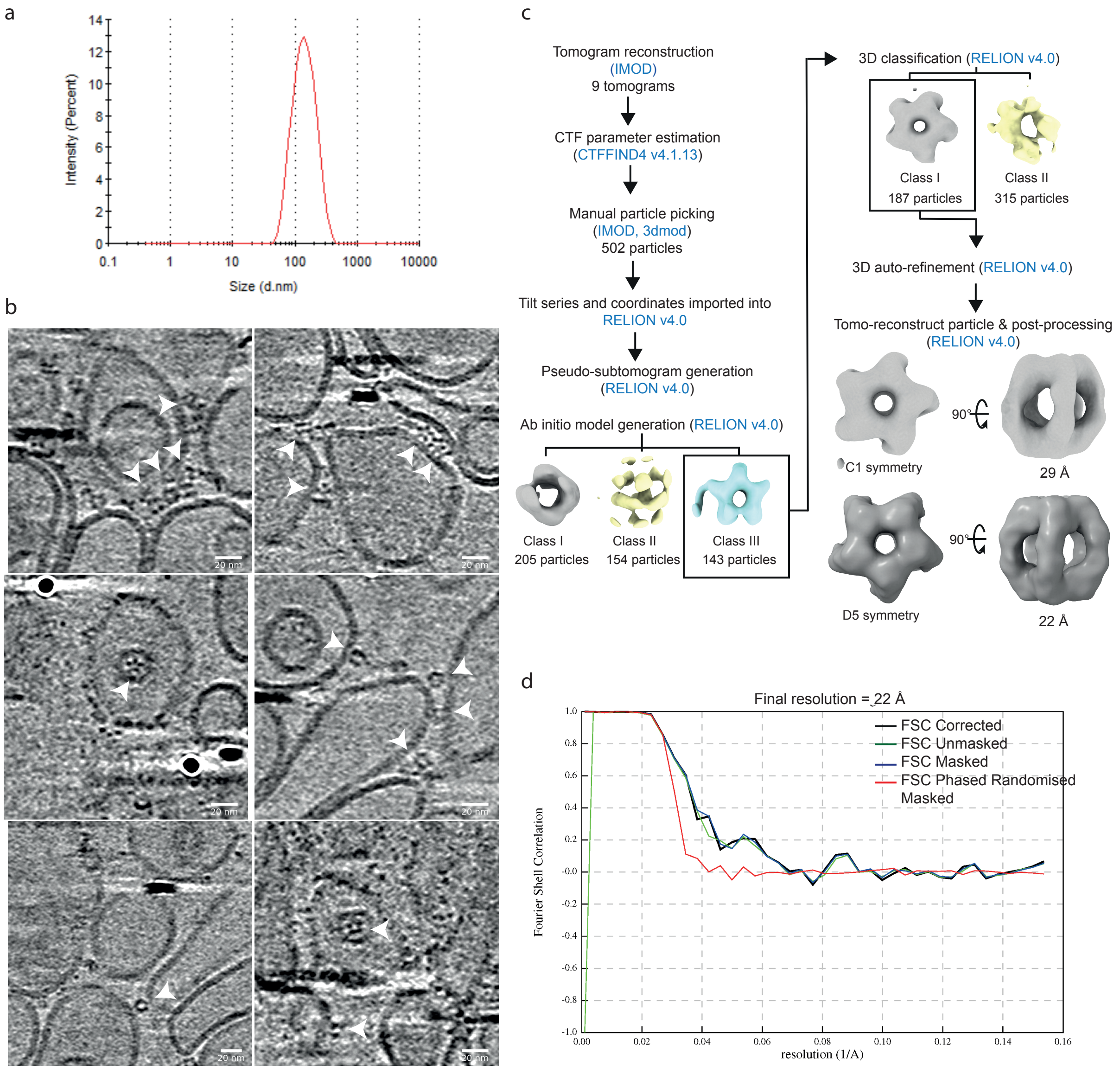

Supplementary Fig. 3. Visualising uncrosslinked GDNF-GFRα1 adhesion complexes on reconstituted liposomes by cryo-ET. (a) Dynamic light scattering size distribution analysis of liposomes prepared using DOPC:DGS-Ni^{2+}-NTA lipids. Shown is a plot of the determined intensity-weighted mean hydrodynamic particle size distribution of DOPC:DGS-Ni^{2+}-NTA liposomes revealing an average liposome diameter of 129.9 d.nm. d.nm = particle diameter in nm. **(b)** 2D tomographic slices from binned by 4 reconstructed tomograms of zGDNF^{mat}-zGFRα1$^{D2\text{-}D3+}$-anchored liposomes. Images show close-up views of bridging protein density between two liposome membranes (indicated with white arrow heads). Two views project down the assembly 5-fold axis. Middle left and bottom right panels. Scale bar: 20 nm. **(c)** Processing pipeline for reconstructing tomograms of zGDNF-zGFRα1 adhering liposomes and sub-tomogram averaging of the zGDNF-zGFRα1 adhesion complex. Particles belonging to the highest resolution class with good quality density (indicated by a black box) were selected during *ab initio* model generation and 3D classification. Numbers below each map indicate the total number of particles contributing to each 3D class average. A map calculated with C1 symmetry indicating the assembly exhibited 5-fold symmetry, therefore the final map was reconstructed with D5 symmetry imposed **(d)** Fourier shell correlation (FSC) curve of the final sub-tomogram

average cryo-EM map the zGDNF-zGFRα1 adhesion complex. Resolution reported based on the gold-standard FSC threshold (FSC = 0.143 criterion).

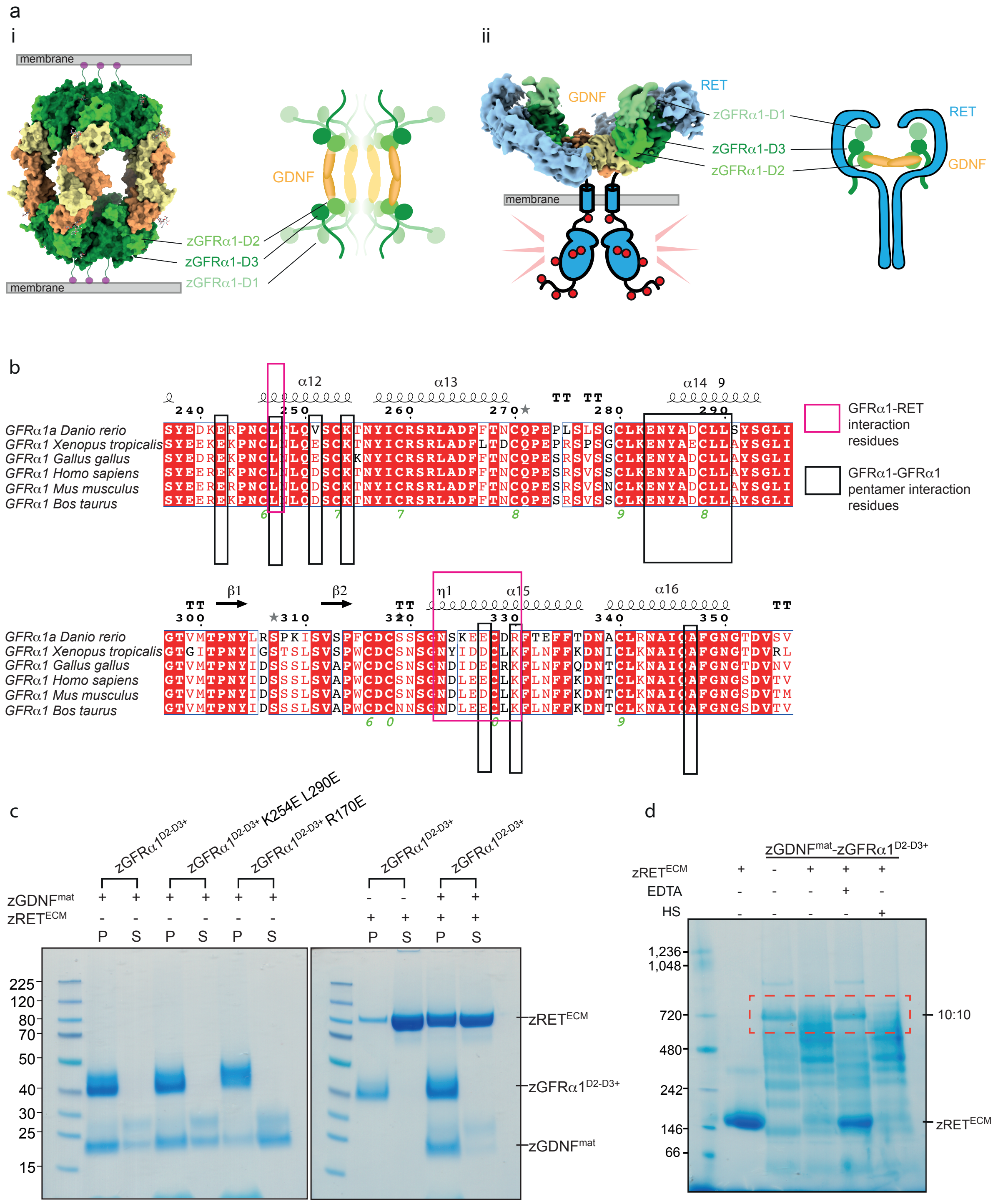

Supplementary Fig. 4. Membrane orientation and competing interfaces within the GDNF-GFRα1 adhesive complex and GDNF-GFRα1-RET trophic complex. (a) (i) zGDNF^{mat}-zGFRα1^{D2-D3+} decamer crystal structure (PDB: 8OS6) mediating adhesion between two opposing membranes. Shown as per Figure 1 with same domain colours. The distance between the membranes corresponds to 15 nm, as estimated from the reported 13.8 nm height of the zGDNF^{mat}-zGFRα1^{D2-D3+} decamer crystal structure. This contrasts with the width of the synaptic cleft reported to be 20 nm by electron microscopy of resin-embedded samples and around 25 nm in cryo-preserved samples. This discrepancy is likely accounted for by the 65 amino acids from the C-terminal of each GFRα1 protomer (shown emanating from the D3 domain

and anchored to the cellular membrane via a GPI-modification) that would be present in the full-length but lacking in the GFRα1^{D2-D3+} construct. ii) Cryo-EM map (EMD-11822) of the reconstituted zGDNF$^{mat}{}_{2}$-zGFRα1$^{D1-D3}{}_{2}$-zRET$^{ECM}{}_{2}$ complex segmented and coloured by protein chain as labelled. The schematic shows the different relative orientation of GDNF with respect to the cellular membrane and GFRα1 D2-D3 domains. **(b)** Sequence alignment of GFRα1^{D2-D3} domains by Espript (http://espript.ibcp.fr)[9] Secondary structure elements from the zebrafish GFRα1^{D1-D3} structure (PDB: 7AML) are annotated above and disulphide linked cysteine pairs indicated by green numbers below. Invariant residues are boxed in red and similar residues in red text. Interaction residues at the GFRα1-RET high affinity site are boxed in pink and interaction residues at the GFRα1:GFRα1 pentamer interface are boxed in black. **(c)** Reducing SDS-PAGE gel of liposome pelleting membrane (P) and soluble fractions (S). His_6-tagged zGFRα1 wild-type and mutant constructs conjugated to the liposome membrane are indicated above each lane and the presence/absence of untagged zGDNF^{mat} and zRET^{ECM} in each sample indicated (+/-). **(d)** Native-PAGE gel of zGDNF^{mat}-zGFRα1^{D2-D3+} in the presence or absence of zRET^{ECM} as indicated. The dashed box indicates the position of the 700 kDa band corresponding to the decameric zGDNF-zGFRα1^{D2-D3+} complex. The first lane is zRET^{ECM} alone. In the presence of zRET^{ECM} the formation of the zGDNF-zGFRα1^{D2-D3+} decamer is disrupted in a Ca^{2+}-dependent manner. Similar results were obtained in two other biological repeats.

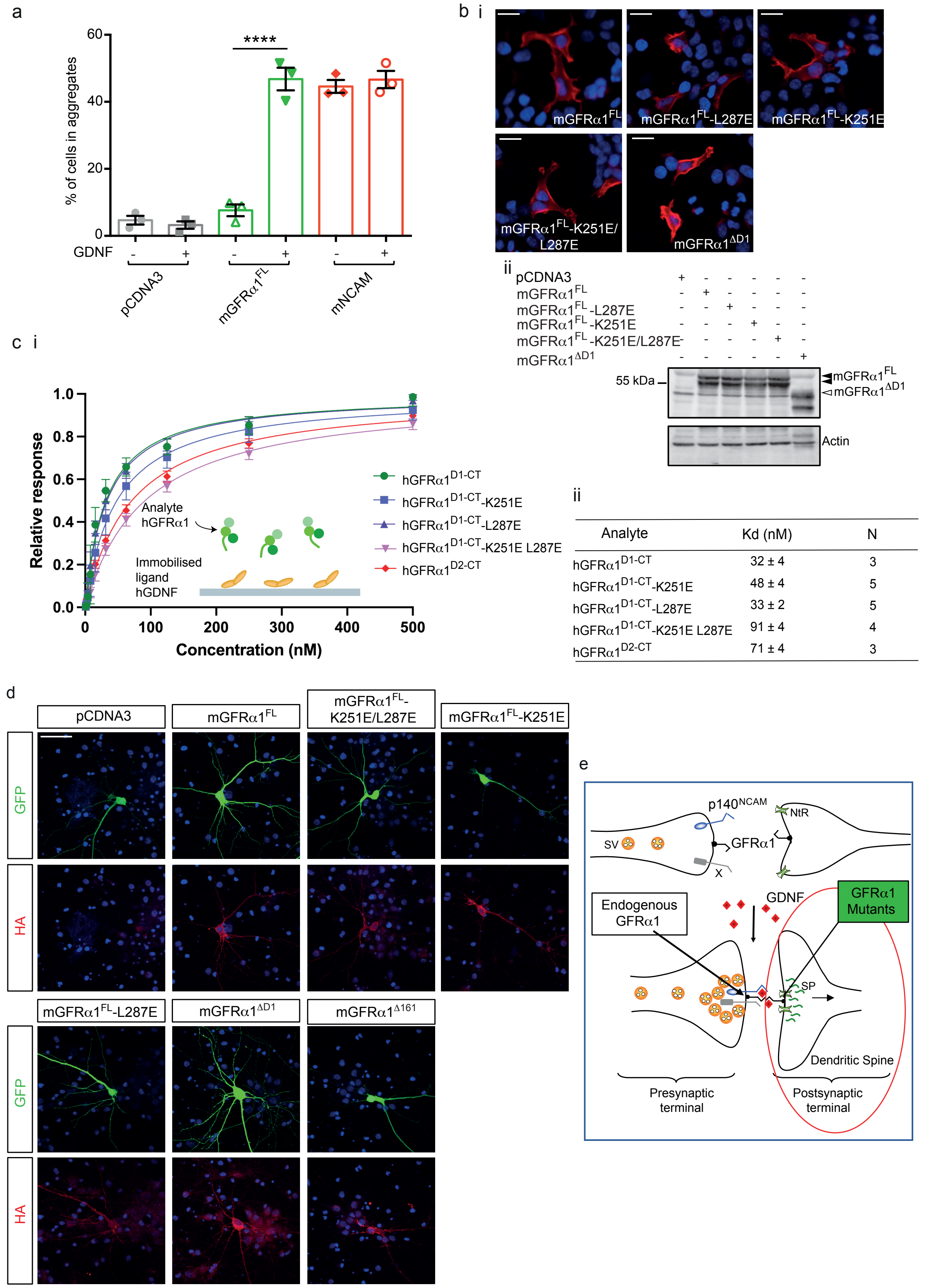

Supplementary Fig. 5. Controls for GDNF-GFRα1-mediated HEK293 cell adhesion and hippocampal synaptogenesis assays. (a) Adhesion capacity of HEK293T cells transiently transfected with mGFRα1^{FL}, mNCAM or pcDNA3 control and GFP ± GDNF. The level of adhesion was evaluated as the percentage of GFP+

cells present in aggregates of 5 or more cells/field. Mean values of triplicate experiments (each measured in duplicate) ± s.e.m. were assessed by one-way analysis of variance (ANOVA), followed by Tukey´s multiple comparison test. ****p < 0.0001 **(b)** Analysis of expression and cellular localization of HA-tagged mGFRα1 mutants in HEK293T cells. i) Immunofluorescence imaging of HEK293T cells transfected with mGFRα1 mutants demonstrate that mutants are correctly localized at the plasma membrane. Scale bar: 10 μm. ii) Immunoblot to show the level of expression of indicated mGFRα1 variants compared to wild-type in HEK293T cells. Both i & ii were stained using anti-HA antibodies (see Supplementary methods). **(c)** SPR analysis showing different mammalian GFRα1 mutants retain full GDNF binding functionality. i) Inset, $hGDNF^{mat}$ was covalently coupled to a CM5 chip and various concentrations of $mGFR\alpha1^{D1\text{-}CT}$ constructs were injected over the chip surface. The maximum response reached for each analyte concentration was fitted to a steady state 1:1 affinity binding model to determine an equilibrium binding constant (K_d) for each construct. ii) Table of determined K_d values for each construct representing the mean value of at least three technical replicates with the SE represented. N value indicates the number of technical repeats. **(d)** Analysis of expression and cellular localization of GFRα1 mutants in hippocampal neurons. Immunofluorescence imaging of hippocampal neurons co-transfected with GFRα1-HA mutants and GFP to show that they are expressed and localized correctly to the plasma membrane. Immunostaining was done with anti-HA antibodies (dil 1:400) on neuronal cultures that were not permeabilized. Nuclei were stained with DAPI. Scale bar: 25 μm. **(e)** Schematic representation of the synaptogenic assay using dissociated hippocampal neurons[10,11], adapted from Paratcha and Ledda., 2008[12]. The consequences of mGFRα1 mutants presented on the postsynaptic membrane (transfected dissociated hippocampal neurons) was analysed by quantifying the dendritic spine density. The presynaptic membrane (axon) expresses endogenous $mGFR\alpha1^{FL}$.

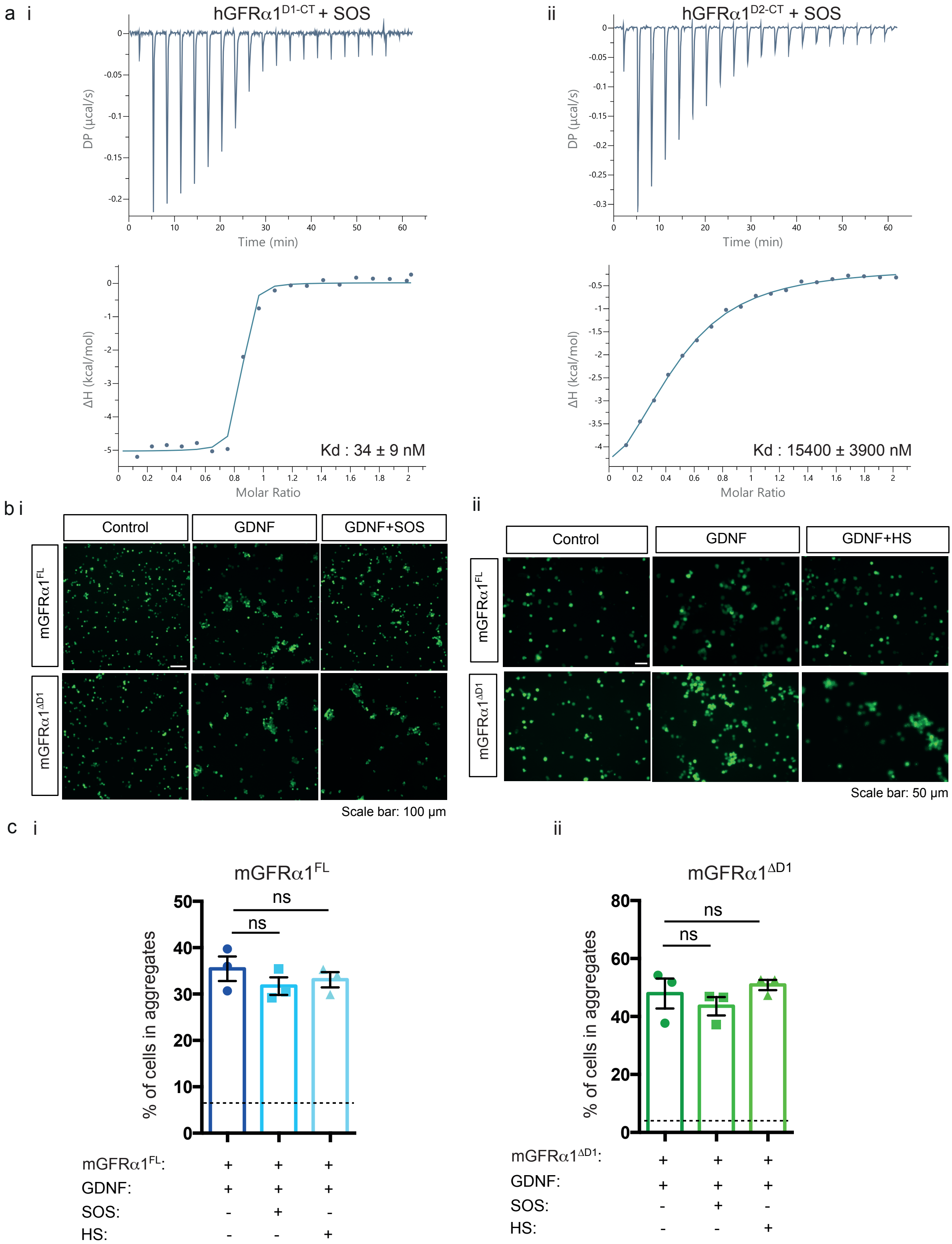

Supplementary Fig. 6. SOS binding to GFRα1 is D1-dependent and GAGs do not impact preformed GFRα1-GDNF cell adhesion complexes. (a) ITC analysis of GFRα1 interactions with SOS. i) hGFRα1$^{D1\text{-}CT}$ binding to SOS and ii) hGFRα1$^{D2\text{-}CT}$ binding to SOS. Raw ITC titration data with the fitted offset subtracted plotted against time (top) and integrated heat signals plotted as a function of molar ratio (bottom). Circles represent the integrated heat of interaction, while blue curves represent the best fit obtained by non-linear least-squares procedures using the 'One set of sites'

model. Representative titrations and binding curves of triplicate measurements are shown. Derived binding constants (Kd) are reported on each plot, as mean values of 3 independent experiments ± standard deviation. **(b)** Confocal images of HEK293 adhesion experiment (**Fig. 5d**) to probe the impact of sulfated GAGs on GFRα1 adhesion capacity in the presence of GDNF. HEK293T cells expressing mGFRα1^{FL} or mGFRα1$^{\Delta D1}$ with GFP were preincubated with (i) SOS or (ii) HS both at 0.5 mg/ml for 2 h followed by 2 h of incubation with GDNF at room temperature. The control sample of transfected cells were not treated with GAG or GDNF. Scale bar :100 μm in (i) and : 50 µm in (ii) images. **(c)** HEK293 adhesion assay to probe the impact of GAGs on <u>preformed</u> GDNF-mGFRα1 assemblies within cell clusters. HEK293T cells were transfected with pCDNA3, mGFRα1^{FL} or mGFRα1$^{\Delta D1}$ with GFP. GFP-expressing cells were then incubated with GDNF (as in Fig. 4b) for 2 h at room temperature. (i) SOS or (ii) HS (0.5 mg/ml) was added for an additional 2 h at room temperature. The percentage of cells in aggregates greater than 5 cells under the indicated conditions is shown. Mean values of triplicate experiments (each measured in duplicated) ± s.e.m. One-way ANOVA, followed by Tukey´s multiple comparison test. The dashed lined indicates the percentage of cell aggregates in the absence of GDNF. ns indicates no statistically significant difference.

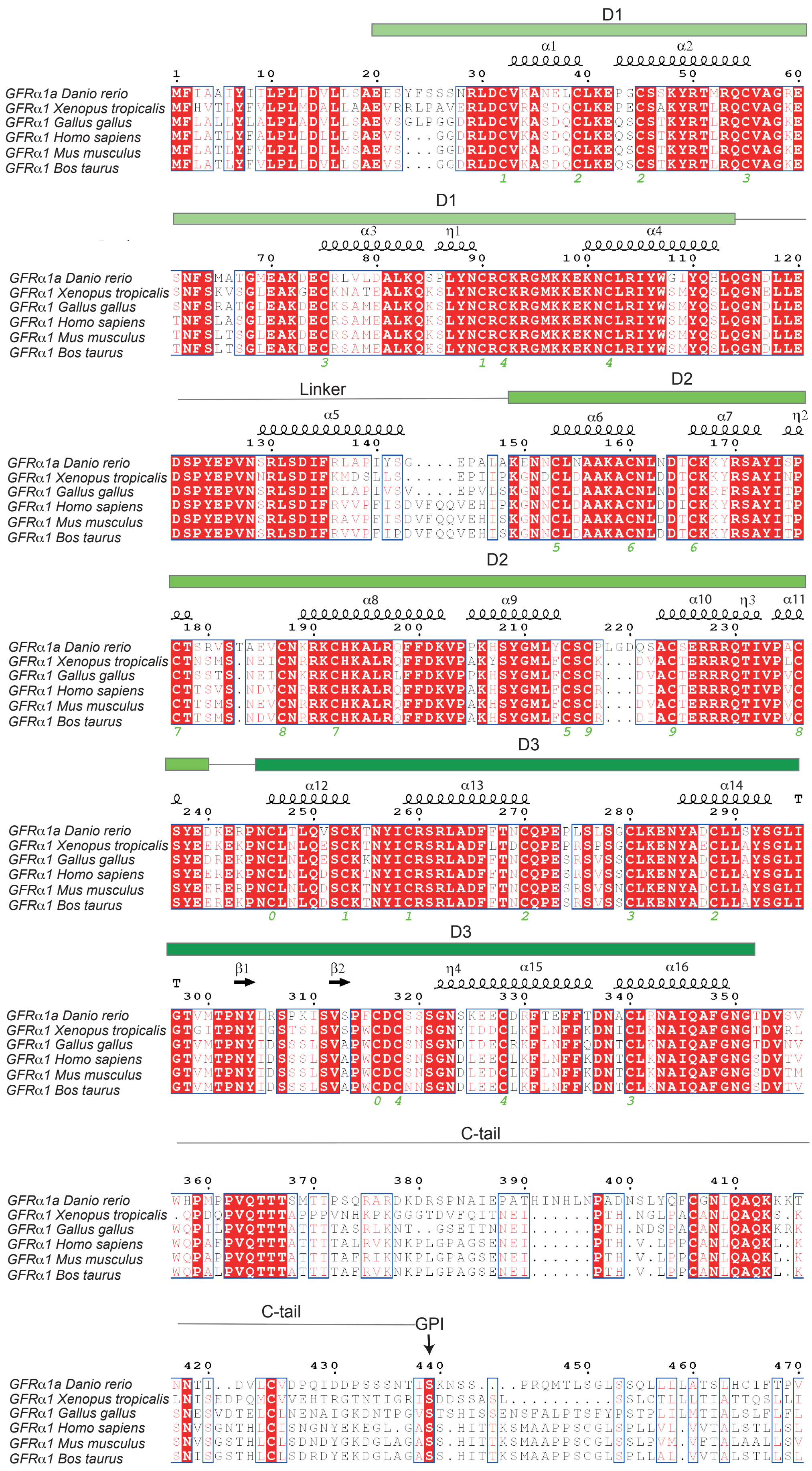
D1
Linker
D2
D3
C-tail
GPI
GFRα1a Danio rerio
GFRα1 Xenopus tropicalis
GFRα1 Gallus gallus
GFRα1 Homo sapiens
GFRα1 Mus musculus
GFRα1 Bos taurus

Supplementary Fig. 7. Multiple sequence alignment for selected species of GFRα1. Sequence alignment of GFRα1 sequences by Espript (http://espript.ibcp.fr)[9]. Secondary structure elements from the zebrafish GFRα1 structure (PDB: 7AML) are annotated above and equivalent numbering is used throughout the text. Disulfide linked cysteine pairs indicated by green numbers below the alignment and domain annotations are shown above the alignment. Invariant residues are boxed in red and similar residues in red text.

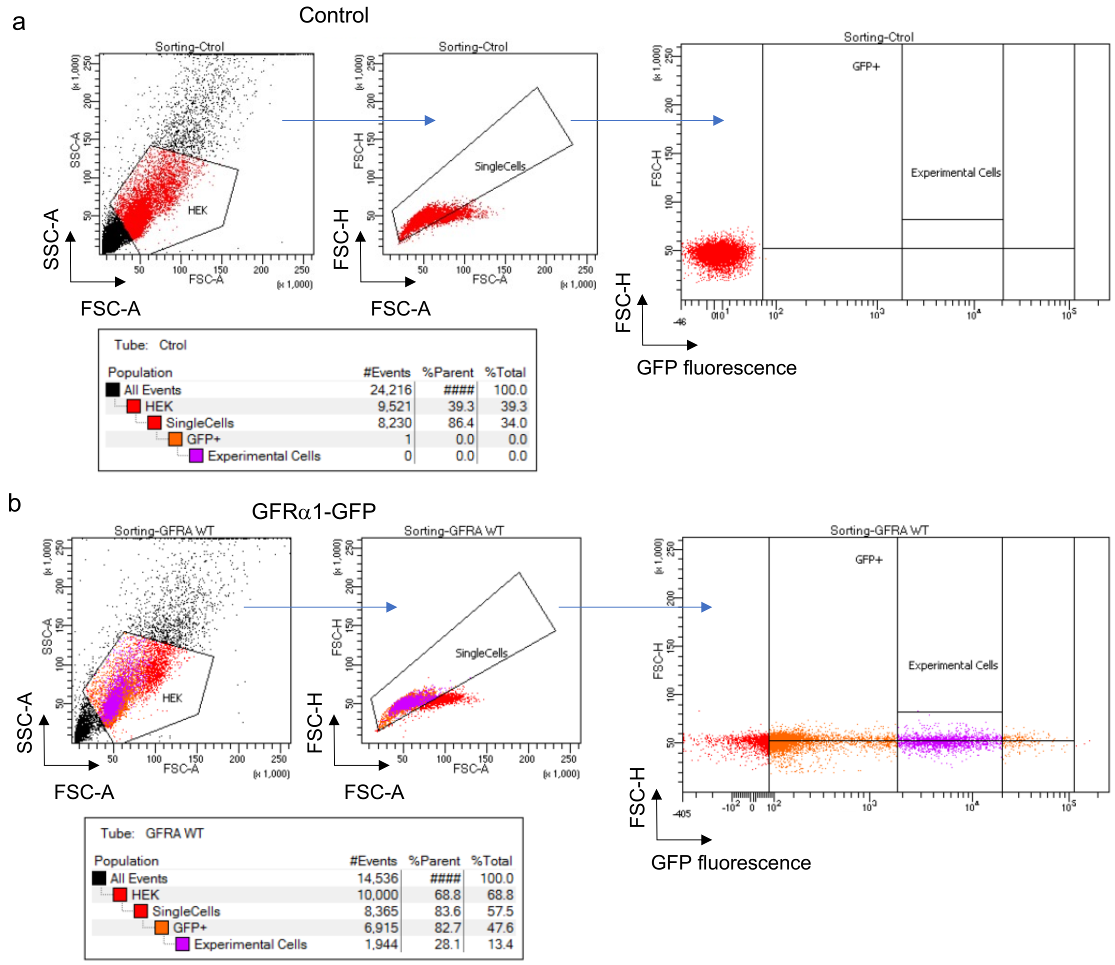

Tube: Ctrol

Population	#Events	%Parent	%Total
All Events	24,216	####	100.0
HEK	9,521	39.3	39.3
SingleCells	8,230	86.4	34.0
GFP+	1	0.0	0.0
Experimental Cells	0	0.0	0.0

Tube: GFRA WT

Population	#Events	%Parent	%Total
All Events	14,536	####	100.0
HEK	10,000	68.8	68.8
SingleCells	8,365	83.6	57.5
GFP+	6,915	82.7	47.6
Experimental Cells	1,944	28.1	13.4

Supplementary Fig. 8 Flow cytometry strategy for cell adhesion assay.
Sorting strategy for live-cells control **(a)** and transfected cells **(b)** using the FACS Aria-Fusion and the FACsDiva versión 8.0.2 software. FSC Area scaling was adjust at 0.45. All live cells were sorted to collect the GFP$^+$ population (purple dots in the graphs). Non-transfected HEK293T cells **(a)** forward vs side scatter (FSC vs SSC) area plot (red population) was analyzed in order to remove debris, followed by a side area vs side height (FSC-A vs FSC-H) to remove doublets. The tables show the percent of total HEK293T cells sorted including the doublets (HEK293T, in red), the percent of single HEK293T cells (Single Cells, in red), the percent of total GFP+ cells (GFP+ cells, in orange) and the percent of GFP+ cells used in the experiment (Experimental cells, in purple).Sorting settings were: nozzle of 85 micrón; precision: Purity; laser: 488 nm.

Assay	Constructs name	Construct details	Expression system	Rationale
Crystal structure	zGDNF^{mat}-zGFRα1^{D1-D3}	zGDNF$^{134-235}$-zGFRα1$^{20-353}$	Sf21 Insect cells	Lacks disordered C-tail and has simple glycans from Sf21 to aid crystallisation.
Native-PAGE	zGDNF^{mat}-zGFRα1^{D1-D3+}<br>zGDNF^{mat}-zGFRα1^{D2-D3+}	zGDNF$^{134-235}$-zGFRα1$^{20-368}$<br>zGDNF$^{134-235}$-zGFRα1$^{144-368}$	Expi293	D1 removed to promote decamer formation. Additional unstructured C-tail residues to improve protein expression.
NS-EM structure	zGDNF^{mat}-zGFRα1^{D2-D3+}	zGDNF$^{134-235}$-zGFRα1$^{144-368}$	Expi293	As above
Liposome aggregation and cryo-ET	zGFRα1^{D2-D3+}<br>zGDNF^{mat}	zGFRα1$^{144-368}$<br>zGDNF$^{134-235}$	Expi293<br>Expi293	As above
Cell adhesion assay	HA-rGFRα1^{FL}<br>HA-rGFRα1$^{\Delta D1}$<br>rGDNF^{mat}	HA-rGFRα1$^{1-468}$<br>HA-rGFRα1$^{154-468}$<br>rGDNF$^{78-211}$	HEK293T<br>HEK293T<br>Sf21 insect cells	Full length and ΔD1 constructs[10]<br>Purchased from R&D
Dendritic spine assay	HA-rGFRα1^{FL}<br>HA-rGFRα1$^{\Delta D1}$<br>rGDNF^{mat}	HA-rGFRα1$^{1-468}$<br>HA-rGFRα1$^{144-468}$<br>rGDNF$^{78-211}$	Rat hippocampal neurons<br>Sf21 insect cells	Rat constructs in rat neurons[11].<br>Purchased from R&D
ITC GFRα1-GAG interactions	hGFRα1^{D1-CT}<br>hGFRα1^{D2-CT}	hGFRα1$^{25-424}$<br>hGFRα1$^{150-424}$	Expi293<br>Expi293	Near full length to improve protein expression of human GFRα1.
SPR GFRα1-GDNF interactions	hGFRα1^{D1-CT}<br>hGDNF^{mat}	hGFRα1$^{25-424}$<br>hGDNF$^{110-211}$	Expi293<br>Expi293	As above<br>Equivalent to zGDNF$^{143-235}$

Supplementary Table 1. Summary of proteins used for each experiment setup used in this study.

Sequence Identity % (Sequence Similarity %)			
	**Zebrafish GDNF^{mat}**	**Human GDNF^{mat}**	**Rat GDNF^{mat}**
**Zebrafish GDNF^{mat}**	100	68 (89)	67 (89)
**Human GDNF^{mat}**		100	94 (99)
**Rat GDNF^{mat}**			100
	**Zebrafish GFRα1^{mat}**	**Human GFRα1^{mat}**	**Rat GFRα1^{mat}**
**Zebrafish GFRα1$^{D1\text{-}D3+}$**	100	63 (82)	63 (82)
**Human GFRα1$^{D1\text{-}D3+}$**		100	94 (99)
**Rat GFRα1$^{D1\text{-}D3+}$**			100

Supplementary Table 2. **Protein sequence identity and similarity between GDNF and GFRα1 sequences.** Human GDNF^{mat} = residues 110-211. Human GFRα1$^{D1\text{-}D3+}$ = residues 25-370

Interaction	K_d (nM)	N (sites)	ΔH (kcal/mol)	ΔG (kcal/mol)	-TΔS (kcal/mol)
**hGFRα1$^{D1\text{-}CT}$ + SOS**	34 ± 9	0.81 ± 0.05	-5.0 ± 0.1	-10.0 ± 0.2	-5.0 ± 0.1
**hGFRα1$^{D2\text{-}CT}$ + SOS**	15400 ± 3900	0.58 ± 0.12	-5.8 ± 1.3	-6.5 ± 0.1	-0.6 ± 1.3
**hGFRα1$^{D1\text{-}CT}$ + HSdp10**	82 ± 49	0.41 ± 0.11	-6.2 ± 1.8	-9.6 ± 0.3	-3.4 ± 1.9
**hGFRα1$^{D2\text{-}CT}$ + HSdp10**	12400 ± 1900	0.50 ± 0.12	-6.5 ± 2.2	-6.6 ± 0.1	-0.1 ± 2.2

Supplementary Table 3. Thermodynamic analysis of GFRα1-GAG interactions by ITC. Table of derived thermodynamic parameters, stoichiometries and binding constants (K_d) obtained for each ITC experiment, given as mean values of at least three independent experiments with standard deviations quoted.

SUPPLEMENTARY METHODS

Surface plasmon resonance (SPR) to measure binding affinity of hGFRα1 mutants to hGDNF

All SPR experiments were performed on a Biacore^{TM} S200 instrument (Cytiva) (v.1.1). Proteins were immobilised on the chip surface using amine coupling according to the manufacturer's protocol (Biacore Handbook, Cytiva). The CM5 (Cytiva) chip surface was activated with 1-ethyl-3-(3-dimethylaminopropyl carbodiimide (EDC)- N-hydroxysuccimide-(NHS) solution (50:50) for 4 min using a flow rate of 10 ul/min at 25 °C. 5 ug/ml hGDNF in 10 mM sodium citrate pH 5.5 was injected over separate activated surfaces resulting in an immobilisation level of 500 response units (RU). The chip surface was subsequently washed with 50 mM NaOH and the remaining free activated carboxyl groups were deactivated with 1M ethanolamine pH 8 for 4 min. A channel surface to be used as a reference was also subjected to activation and deactivation (control flow cell).

The interaction analysis was performed in a running buffer 20 mM HEPES pH 7.5, 130 mM NaCl, 0.05 % Tween at 25 °C with a flow rate of 30 µl/min. hGFRα1 analytes were diluted by a serial two-fold dilution series in running buffer and injected alongside a blank sample over immobilised protein and the reference surface for 240 s followed by 300 s of dissociation. Each reaction surface was regenerated by injecting 1M ethanolamine pH 8 for 30 s between analyte injections. Injection series were repeated in at least triplicate, exact repeat numbers are indicated in Supplementary Fig. 5 c i.

Final sensorgrams were generated by subtracting the reference (from control flow cell) and blank (solvent injection) sensorgrams from the reaction flow cell sensorgram. The normalised maximum response for each analyte concentration value at equilibrium was plotted as a function of analyte concentration. Each data point represents the average values from at least three independent experiments, with error bars showing standard deviation from the mean. Maximum response data was fitted to a 1:1 ligand to analyte steady state affinity binding model to determine equilibrium binding constants. Data analysis was carried out using the Biacore^{TM} S200 Evaluation

Software (Cytiva) (v.1.1) and GraphPad Prism9 (GraphPad Software, San Diego, California USA, www.graphpad.com).

Liposome pelleting assay

For liposome pelleting assay, lipids (3 mM) and each protein sample (3 μM) were mixed in a final volume of 300 μl of assay buffer containing 20 mM HEPES (pH 7.5), 180 mM NaCl ± 1mM $CaCl_2$ or 1 mM EDTA as indicated. Samples were incubated for 30 min at room temperature before being centrifuged at 200,000 x g for 50 min, 4 °C. The supernatant was removed and the lipid membrane pellet resuspended in assay buffer + 1 % Triton X 100. 30 μl of supernatant (S) and pellet (P) fractions were loaded onto a reducing SDS-PAGE gel to image protein partitioning.

Immunofluorescence

Transfected HEK293T cells or hippocampal neurons were fixed with 4% PFA followed by immunofluorescence using anti-HA (Clone 12CA5; dilution 1:400) from Roche (cat#11666606001). Secondary antibodies were from Jackson ImmunoResearch: Cy3-Donkey anti-Mouse IgG (H+L) (dil 1:200, cat#715-165-150).

Immunoblot analysis

Western blot analysis of rGFRα1-HA expression was performed as previously described (Ledda et al., 2007)[10]. Briefly, transfected HEK293T cells were homogenized in ice-cold 25 mM Tris-HCl (pH 7.4), 1 mM EDTA and 125 mM NaCl, 0.5% Triton and protease inhibitors (Roche, cat# 0505648900). After centrifugation at 10,000 x g 20 min, the supernatant was analysed by western blot to evaluate the protein levels of rGFRα1-HA. Antibodies: anti-HA (Clone 12CA5, dilution 1:800) from Roche (cat#11666606001), anti-actin H-6 (dilution 1:6,000) from Santa-Cruz (cat#sc-376421) and the secondary antibody from Jackson ImmunoResearch: Alkaline Phosphatase-Donkey anti Mouse IgG (H+L) (1:10,000) were used. Immunoblots were scanned in a Storm 845 PhosphorImager (GE Healthcare Life Sciences).

SUPPLEMENTARY REFERENCES

1. Vagin, A. & Teplyakov, A. MOLREP: an Automated Program for Molecular Replacement. *J Appl Crystallogr* **30**, 1022–1025 (1997).
2. Potterton, E., Briggs, P., Turkenburg, M. & Dodson, E. A graphical user interface to the CCP4 program suite. *Acta Crystallogr D Biol Crystallogr* **59**, 1131–1137 (2003).
3. Agirre, J. *et al.* Privateer: Software for the conformational validation of carbohydrate structures. *Nat Struct Mol Biol* **22**, 833–834 (2015).
4. Schrödinger: The PyMOL molecular graphics system - Version 2.4. (2020).
5. Parkash, V. & Goldman, A. Comparison of GFL-GFRalpha complexes: further evidence relating GFL bend angle to RET signalling. *Acta Crystallogr Sect F Struct Biol Cryst Commun* **65**, 551–558 (2009).
6. De la Rosa-Trevín, J. M. *et al.* Xmipp 3.0: An improved software suite for image processing in electron microscopy. *J Struct Biol* **184**, 321–328 (2013).
7. Scheres, S. H. W. RELION: Implementation of a Bayesian approach to cryo-EM structure determination. *J Struct Biol* **180**, 519–530 (2012).
8. Zivanov, J. *et al.* New tools for automated high-resolution cryo-EM structure determination in RELION-3. *Elife* **7**, e42166 (2018).
9. Robert, X. & Gouet, P. Deciphering key features in protein structures with the new ENDscript server. *Nucleic Acids Res* **42**, W320–W324 (2014).
10. Ledda, F., Paratcha, G., Sandoval-Guzmán, T. & Ibãez, C. F. GDNF and GFRα1 promote formation of neuronal synapses by ligand-induced cell adhesion. *Nature Neuroscience 2007 10:3* **10**, 293–300 (2007).
11. Irala, D. *et al.* The GDNF-GFRα1 complex promotes the development of hippocampal dendritic arbors and spines via NCAM. *Development* **143**, 4224–4235 (2016).
12. Paratcha, G. & Ledda, F. GDNF and GFRα: a versatile molecular complex for developing neurons. *Trends Neurosci* **31**, 384–391 (2008).
